# Anatomical Evaluation of Mandibular Molars in a Saudi Population: An In Vivo Cone-Beam Computed Tomography Study

**DOI:** 10.1155/2021/5594464

**Published:** 2021-03-30

**Authors:** Mohammed Mashyakhy, Ahmad Jabali, Fatimah Saleem Alabsi, Abdulaziz AbuMelha, Mazen Alkahtany, Shilpa Bhandi

**Affiliations:** ^1^Department of Restorative Dental Science, College of Dentistry, Jazan University, Jazan, Saudi Arabia; ^2^Department of Restorative Dental Sciences, College of Dentistry, King Khalid University, Abha, Saudi Arabia; ^3^Department of Restorative Dental Sciences, College of Dentistry, King Saud University, Riyadh, Saudi Arabia

## Abstract

**Objective:**

This study assessed the canal configuration of mandibular molars according to Vertucci's classification of a Saudi population using cone-beam computed tomography (CBCT).

**Methods:**

A total of 290 first and 367 second mandibular molars were analyzed. The CBCT images were evaluated in three sections to inspect the number of roots and canals and canal system. The data are presented as frequencies and percentages. The chi-squared test was used to assess differences between both sides. SPSS was used for analysis, with a significance level of *α* ≤ 0.05.

**Results:**

Among the first molars, 95.4% of the teeth had two roots, and 64.5% had three canals. Approximately 57.9% showed Vertucci type IV mesial roots. Between the second molars, 89.6% of teeth had two roots and 80.4% had three canals. The frequency of Vertucci type IV (39.4%) was the highest in mesial roots. The first molars showed a high prevalence of 3-rooted teeth (5.5%). Both the right and left sides showed teeth with similar external and internal anatomy (*p* < 0.05).

**Conclusion:**

Most of the mandibular first and second molars had two roots and three canals. In the first mandibular molars, similar to the second mandibular molars, the majority of the mesial canals had Vertucci type IV, while the distal canals had Vertucci type I.

## 1. Background

The success of a root canal treatment (RCT) is highly dependent on adequate knowledge and understanding of the external and internal morphology of the root canal system (RCS), which permits proper chemomechanical cleaning and shaping of all the pulp spaces [1].

Clinicians should be aware of the possible variations in root and root canal morphology and employ the available diagnostic tools to avoid missing some RCS anatomy. Mandibular molars are characterized by the presence of two roots (mesial and distal); however, the prevalence of three-rooted mandibular molars is common [2, 3]. A complex morphology of the RCS may be encountered during the treatment of these teeth [4, 5]. Preoperative diagnosis is among the key aspects promoting a good prognosis of the endodontic treatment performed [6]. Preoperative endodontic imaging aids in assessing the root morphology, root size, internal structure, and periapical condition of the offending tooth. In the absence of radiographic images, pathology identification and treatment become perplexing [7]. Pretreatment evaluation reveals the challenges that the dentist may be faced with during treatment, such as calcified, tortuous, and intricate canal anatomy, in addition to the proximity of a pathology surrounding anatomic structures. For accurate analysis and treatment planning, the area of concern must be properly selected to determine supplementary investigations or further treatment plan [8].

Researchers have proposed numerous methods to study the anatomy of the tooth, such as various radiographs before treatment with varying angulations and analysis [9], staining techniques [10], tomography [11], and radiography using contrast media [12]. Some problems related to conventional radiography include superimposition of the adjacent anatomic structure, noise, and geometric distortion [13–15].

The advent of cone-beam computed tomography (CBCT) in dentistry offers a three-dimensional (3D) view of the oral and maxillofacial structures providing a characteristic diagnostic aid [16]. Owing to the minimal radiation exposure when compared to traditional CT, CBCT has a prime role in pathology identification and treatment planning in endodontics [17, 18]. The dawn of effortlessly available, high-spatial-resolution CBCT conveys a lower radiation dose than the emission amount compared to a multidetector computed tomography and delivers endodontists with a feasible cross-sectional imaging modality. The CBCT's multiplanar evaluation of the structure eliminates superimposition and anatomical noise [19].

In the literature search in a Saudi population, a few anatomical studies utilizing different methodologies have been performed on mandibular molars [2, 5, 20–24] with limitations including not exploring all aspects of morphology and emphasis on C-shaped canal configurations and extra rooted molars only. Thus, the present in vivo CBCT study was designed to evaluate the number of roots and root canals and internal morphology and compare the right and left permanent mandibular first and second molars.

## 2. Materials and Methods

The database archive of the College of Dentistry at Jazan University was searched for CBCT scans available from 2016 to 2017. A retrospective study consisting of 208 CBCT scans was performed. These radiographs included those of 100 males and 108 females in the age group ranging from 17 to 59 years. Scans were previously taken in different departments for diagnostic and treatment planning purposes. Ethical approval was obtained from the Standing Committee for Scientific Research Ethics at Jazan University (Reference no. REC41/4/087). The CBCT device utilized for research was a three-dimensional Accuitomo 170 (MORITA, Japan) with the following parameters: 5–8 mA, 90 kv, with 17.5 s exposure time and 0.25 mm voxel size and Morita's i-Dixel 3D software imaging program was used for the processing of the CBCT radiographs.

A total of 657 mandibular first and second molars (290 first and 367 second molars) were analyzed in the current study. Teeth were included if they had fully developed roots and closed apices. The exclusion criteria were teeth with root canal treatment, calcified canals, and resorbed roots. Distorted or unclear teeth on CBCT scans were excluded. Three sections were acquired for each tooth (serial axial, coronal, and sagittal) to ensure the accuracy of the evaluation of the teeth. All the teeth were included in the evaluation of the external morphology. For internal morphology, however, teeth with a C-shaped configuration were excluded. The evaluated parameters were the number of roots, number of root canals, and root canal configurations according to Vertucci's taxonomy [25]. A comparison between the right and left sides was also performed.

To reduce measurement errors, one observer evaluated 25% of the samples twice with an interval period of 3 weeks. The Kappa test results revealed an almost impeccable concordance between interpretations with a value of 88.4% and asymptotic standard error of ±9.5%.

### 2.1. Statistical Analysis

The Statistical Package for Social Sciences software program for Windows (SPSS V25; IBM, Chicago, IL, USA) was used for data analysis. In addition to the frequencies and percentages, the chi-squared test was used to determine the differences between the right and left sides. To compare variables with more than two categories, the contingency coefficient option was selected. The significance level was set at *p* < 0.05 for all analyses.

## 3. Results

**Figure 1:**
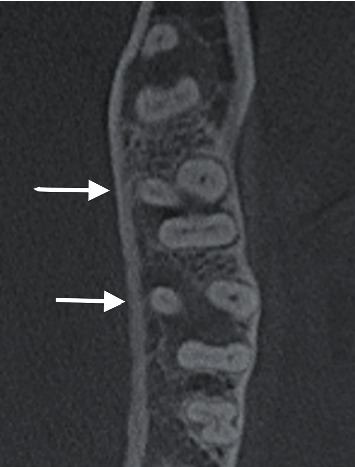
CBCT axial section at midroots of first and second mandibular molars showing the presence of a third root (arrows).

**Figure 2:**
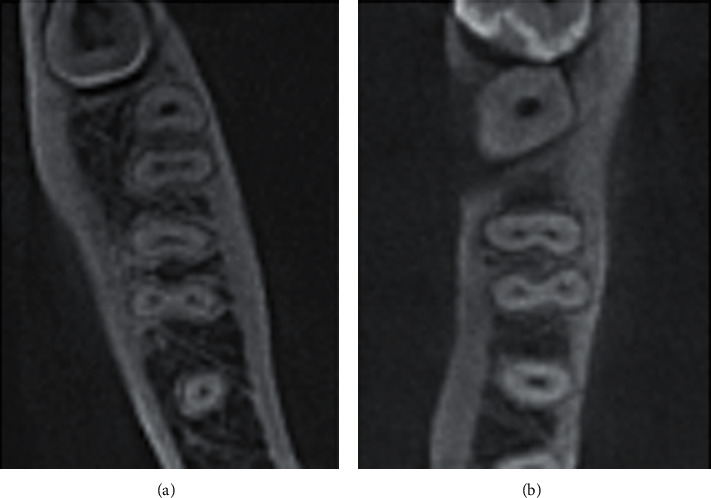
CBCT axial sections at midroots. (a) first and second mandibular molars with three canals and (b) first mandibular molar with four canals.

**Figure 3:**
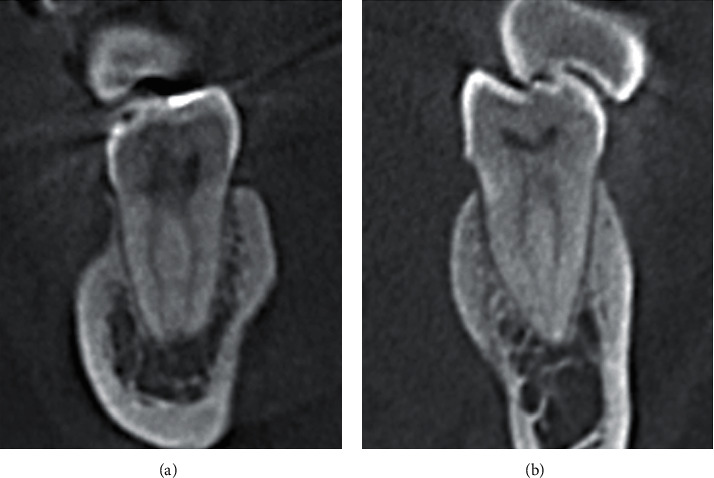
CBCT coronal sections. (a) Vertucci type IV canal configuration in mesial root of a mandibular first molar and (b) Vertucci type II in distal root of a mandibular first molar.

In this study, 290 mandibular first molars were assessed (Table 1). The majority (94.5%) had two roots, and 16 (5.5%) teeth had three roots, in which the extra roots were all lingual (Figure 1). A total of 187 (64.5) teeth had three canals, and 101 (34.8%) teeth had four canals (Figure 2). More than half of the sample (57.9%) had Vertucci type IV in the mesial canals. In contrast, 105 teeth (36.2%) had Vertucci type II canals. In contrast, 200 (69.0%) teeth had Vertucci type I and 50 (17.2%) teeth had Vertucci type III in the distal canals (Figure 3).

No significant difference was observed in the number of roots (*p*=1.000), the number of canals (*p*=0.361), and canal configurations in mesial roots (*p*=0.312) and distal roots (*P*=0.978) between the right and left sides. Additional details are provided in Table 2.

A total of 367 mandibular second molars were evaluated (Table 3). Of them, 329 (89.6%) teeth had two roots, 31 (8.5%) molars had one root (fused roots), and seven (1.9%) teeth had three roots. Regarding the internal morphology, 295 (80.4%) teeth had three canals, 23 (6.3%) teeth had two canals, and 20 (5.4%) teeth had four canals. Among the teeth with fused roots, 29 teeth (7.9%) displayed other canal configurations. The frequency of Vertucci type IV (39.4%) was the highest in mesial canals followed by Vertucci type II (25.4%). In contrast, Vertucci type I was the most frequent type in the distal canals (95.6%) and Vertucci type IV was not detected.

The distribution of the number of roots and the number of canals on both sides was nearly similar, with no significant difference (*p*=0.935 and *p*=0.575, resp.) and the same for both mesial (*p*=0.780) and distal (*p*=0.857) roots based on Vertucci's classification. Additional details are provided in Table 4.

## 4. Discussion

In the current study, CBCT was used to study the root canal anatomy of mandibular first and second molars. The mandibular first molar is among the first permanent teeth to erupt in the oral cavity. It is most prone to caries owing to its immature enamel since its phase of mineralization concurs with early childhood caries and the window of infectivity. It erupts when the maintenance of oral hygiene is challenging, thus making it more susceptible to dental caries [26]. Owing to the complex root canal morphology of mandibular molars, especially in the mesial root, root canal therapy is a challenging task with an unexpected prognosis. The mesial root of the mandibular first molar is wide buccolingually and narrow mesiodistally. In comparison, the distal root is conical in shape [27]. The mandibular second molars mostly shared the same characteristics.

The root canal anatomy of the teeth has been discussed, researched, and categorized by many scholars based on the number of roots and root canals in the teeth. Weine et al. first classified the canal configuration of teeth with one root into four basic types [28]. However, this classification is not applicable to teeth with multiple roots. In 1984, Vertucci published a new classification by expanding Weine's classification and using it to classify maxillary first premolars. He added four additional types of configurations to Weine's classification [25]. Recently, Ahmed et al. [29, 30] introduced a new classification system based on 3D technology to describe quickly and precisely the RCS that could be applicable in research, clinical practice, and training. Despite certain drawbacks, Vertucci's classification is the most commonly used classification system to date [31]. In the present study, Vertucci's classification was used to study the root canal configuration in the Saudi population.

CBCT is the best imaging technique for the analysis of root canal morphology owing to its diagnostic accuracy and feasibility [16, 32, 33]. In the present study, CBCT was used to determine the number of roots, the number of root canals, and the internal canal configuration of the mandibular first and second molars. A comparison was also made between the contralateral mandibular molars.

The majority of mandibular first and second molar teeth presented with two roots (94.5% and 89.6%, resp.), similar to previous studies from different populations [3, 34–36]. In addition, 3-rooted molars were observed in higher incidence in the first molars (5.5%) than in the second molars (1.7%), consistent with a recent report on the Saudi population (3.05% and 1.48% for first and second molars, resp.) [2]. Other studies in different populations have shown a similar lower prevalence of extra rooted molars [34, 37]. However, in contrast, other studies in Asian populations showed a high number of extra rooted first molars: 21.6% in Malaysian [38] and 32.35% in Shanghai Chinese populations [39]. Therefore, the prevalence seems to be influenced by ethnicity; complex anatomy is expected in Asian origin.

In the current study, 34.8% of the molars had four canals. It was observed that 31.8% of the two-rooted first mandibular molars had four canals, and 87.5% of the three-rooted mandibular first molars had four canals. In contrast, 64.5% of teeth had three canals, of which 67.5% and 12.5% were in two-and in three-rooted molars, respectively (Table 1). This implies that most of the first molars in the Saudi population have three canals. This finding is in agreement with the research conducted by Mashyakhy et al. on the Saudi population [20], Abarca et al. on the Palestinian population [36], and Senan et al. on the Yemeni subpopulation [40]. In another study published by Silva et al. [41] on CBCT images of mandibular first and second molars of a Brazilian population, the first molars had two separate roots (74%) with a greater occurrence of two canals in the mesial root and one canal in the distal root. In contrast, the mandibular second molars had two distinct roots with two canals; one canal in the mesial root and one in the distant root formed 54% of the sample size.

Additionally, in our study, it was observed that the canal configuration in the mesial root of the mandibular first molar was Vertucci's type IV (57.9%) and that of the distal canal Vertucci's type I (69%). This finding was in accordance with Zhang et al., in which 81% of the mesial canals were Vertucci's type IV, and 84% of canals in distal canals were type I in the Chinese population [42]. However, our findings differed from the findings in the Yemeni population reported by Senan et al., where two roots and three canals were found in 89.4% of teeth, of which, in the mesial canal, 57% were type II and 35.6% were type IV. In a study published by Kantilieraki et al., the mesial canal in the mandibular first molar in the Greek population, 69.8% had a type II configuration, and the distal canal had a type I configuration in 81.7% [3]. In the study published by Torres et al., in Belgian and Chilean populations, 42.8% of the mesial canal in the mandibular first molar had a type V configuration, 33.5% had a type III configuration in the Belgian population, 28.9% had type III canals, and 18.9% had type II configuration in the Chilean population. In comparison, 72.8% of the distal canals had a type I configuration in the Belgian population and 78.8% had a type I population in the Chilean population [35].

In mandibular second molars, it was observed that, in most of the studies, two roots and three canals were more common, which was similar to our study wherein 89.4% of the 2^nd^ molars had two roots and three canals with the structure of mesial canals being similar to Vertucci's type IV in 39.5% of teeth, and 25.5% of teeth had Vertucci's type II configuration. In the distal root, the canals had Vertucci type II (28.6%), type IV (28.6%), and type V (28%) canals. A few teeth were also reported to have three roots with four canals (Table 3). In comparison to other studies, our outcomes were similar to those reported by Zhang et al. on mandibular first and second molars in the Chinese population. He reported that the majority of the mandibular second molars had two roots and three canals, with 57% of the mesial roots having type IV configuration and 97% of the distal canal having a type I configuration [42]. In a study published by Kantilieraki et al., 64.1% had a type II configuration in the mesial canal and the distal canal had a type I configuration in 97.7% in mandibular second molars in the Greek population [2]. In the study published by Torres et al., in Belgian and Chilean populations, 37.2% of the mesial roots of mandibular second molars had a type III configuration, 28.7% had a type V configuration in the Belgian population, 48.4% had type III canals, and 20.6% had type V configuration in the Chilean population. In comparison, 98.9% of the distal canals had a type I configuration in the Belgian population, and 98.9% had a type I population in the Chilean population [35].

In the present study, the right and left sides of mandibular first and second molar anatomy were compared, and no significant differences were observed in the number of roots, number of root canals, and canal configurations of the mesial and distal roots (Tables 2 and 4). Our results are concurrent with a previous report on the Malaysian population using the same methodology [36]. Differences in side help the clinician predict the anatomy of the contralateral tooth when treating both sides.

## 5. Conclusion

Based on the findings of the present study, we conclude that the majority of the first and second mandibular molars have two roots and three canals and the presence of a third root is not uncommon. The configuration of the canals differs from population to population and depends on various factors such as race, genetics, and ethnicity. The configuration of the canals should be determined prior to treatment using CBCT for a better understanding of the root canal system.

## Figures and Tables

**Table 1:** Frequency of number of roots, number of canals, and Vertucci type among mandibular 1^st^ molars *N* (%).

	All teeth (*N* = 290)	2-rooted teeth (*N* = 274)	3-rooted teeth^*∗*^ (*N* = 16)
*Number of canals*			
2 canals	2 (0.7)	2 (0.7)	—
3 canals	187 (64.5)	185 (67.5)	2 (12.5)
4 canals	101 (34.8)	87 (31.8)	14 (87.5)

*M. root Vertucci types*			
Type I	3 (1)	2 (0.7)	1 (6.3)
Type II	105 (36.2)	100 (36.5)	5 (31.3)
Type III	4 (1.4)	3 (1.1)	1 (6.3)
Type IV	168 (57.9)	160 (58.4)	8 (50.0)
Type V	10 (3.4)	9 (3.3)	1 (6.3)

*D. root Vertucci types*			
Type I	200 (69)	184 (67.2)	16 (100.0)
Type II	9 (3.1)	9 (3.3)	—
Type III	50 (17.2)	50 (18.2)	—
Type IV	2 (0.7)	2 (0.7)	—
Type V	29 (10)	29 (10.6)	—

^*∗*^All extra roots had one canal and Vertucci type I.

**Table 2:** Comparison amongst right and left edges among mandibular 1^st^ molars *N* (%).

	All teeth (*N* = 290)			2-rooted teeth (*N* = 274)			3-rooted teeth (*N* = 16)		
	Right	Left	*P*	Right	Left	*P*	Right	Left	*P*
*Number of canals*									
2 canals	2 (1.3)	0 (0.0)	0.361	2 (1.4)	0 (0.0)	0.352	—	—	1.000
3 canals	95 (62.9)	92 (66.2)		94 (65.7)	91 (69.5)		1 (12.5)	1 (12.5)	
4 canals	54 (35.8)	47 (33.8)		47 (32.9)	40 (30.5)		7 (87.5)	7 (87.5)	
Total	151 (52.1)	139 (47.9)		143 (52.2)	131 (47.8)		8 (50.0)	8 (50.0)	

*M. root Vertucci types*									
Type I	3 (2.0)	0 (0.0)	0.312	2 (1.4)	0 (0.0)	0.581	1 (12.5)	0 (0.0)	0.448
Type II	51 (33.8)	54 (38.8)		49 (34.3)	51 (38.9)		2 (25.0)	3 (37.5)
Type III	1 (0.7)	3 (2.2)		1 (0.7)	2 (1.5)		0 (0.0)	1 (12.5)
Type IV	91 (60.3)	77 (55.4)		86 (60.1)	74 (56.5)		5 (62.5)	3 (37.5)
Type V	5 (3.3)	5 (3.6)		5 (3.5)	4 (3.1)		0 (0.0)	1 (12.5)
Total	151 (52.1)	139 (47.9)		143 (52.2)	131 (47.8)		8 (50.0)	8 (50.0)

*D. root Vertucci types*									
Type I	102 (67.5)	98 (70.5)	0.978	94 (65.7)	90 (68.7)	0.980	8 (100.0)	8 (100.0)	NC
Type II	5 (3.3)	4 (2.9)		5 (3.5)	4 (3.1)		—	—
Type III	28 (18.5)	22 (15.8)		28 (19.6)	22 (16.8)		—	—
Type IV	1 (0.7)	1 (0.7)		1 (0.7)	1 (0.8)		—	—
Type V	15 (9.9)	14 (10.1)		15 (10.5)	14 (10.7)		—	—
Total	151 (52.1)	139 (47.9)		143 (52.2)	131 (47.8)		8 (50.0)	8 (50.0)

**Table 3:** Frequency of number of roots, number of canals, and Vertucci type among mandibular 2^nd^ molars *N* (%).

	All teeth (*N* = 367)	1-rooted teeth^†^ (fused; *N* = 31)	2-rooted teeth (*N* = 329)	3-rooted teeth^*∗*^ (*N* = 7)
*Number of canals*				
2 canals	23 (6.3)	1 (3.2)	22 (6.6)	—
3 canals	295 (80.4)	1 (3.2)	294 (89.4)	—
4 canals	20 (5.4)	—	13 (4.0)	7 (100.0)
Other	29 (7.9)	29 (93.6)	—	—

*M. Vertucci types*				
Type I	21 (6.2)	—	21 (6.4)	—
Type II	86 (25.4)	—	84 (25.5)	2 (28.6)
Type III	54 (16)	—	53 (16.1)	1 (14.3)
Type IV	133 (39.4)	1 (50.0)	130 (39.5)	2 (28.6)
Type V	44 (13)	1 (50.0)	41 (12.5)	2 (28.6)

*D. Vertucci types*				
Type I	323 (95.6)	2 (100.0)	315 (95.7)	6 (85.7)
Type II	3 (0.9)	—	3 (0.9)	—
Type III	3 (0.9)	—	3 (0.9)	—
Type IV	—	—	—	—
Type V	9 (2.6)	—	8 (2.4)	1 (14.3)

^†^29 teeth had joined root canal system; they were excluded from internal morphology analysis. ^*∗*^All extra roots had one canal and Vertucci type I.

**Table 4:** Comparison amongst right and left edges among mandibular 2^nd^ molars *N* (%).

	All teeth (*N* = 338)			1-rooted teeth (*N* = 2)			2-rooted teeth (*N* = 329)			3-rooted teeth (*N* = 7)		
	Right	Left	*P*	Right	Left	*P*	Right	Left	*P*	Right	Left	*P*
*Number of canals (N* *=* *338)*												
2 canals	9 (5.4)	14 (8.2)	0.575	1 (100.0)	0 (0.0)	1.000	8 (4.9)	14 (8.5)	0.414	—	—	NC
3 canals	149 (88.7)	146 (85.9)		0 (0.0)	1 (100.0)		149 (90.9)	145 (87.9)		—	—
4 canals	10 (6.0)	10 (5.9)		—	—		7 (4.3)	6 (3.6)		3 (100.0)	4 (100.0)
Total	168 (49.7)	170 (50.3)		1 (50.0)	1 (50.0)		164 (49.8)	165 (50.2)		3 (42.9)	4 (57.1)

*M. Vertucci types (N* *=* *338)*												
Type I	8 (4.8)	13 (7.6)	0.78	—	—	1.000	8 (4.9)	13 (7.9)	0.792	—	—	0.405
Type II	41 (24.4)	45 (26.5)		—	—		40 (24.4)	44 (26.7)		1 (33.3)	1 (25.0)
Type III	29 (17.3)	25 (14.7)		—	—		28 (17.1)	25 (15.2)		1 (33.3)	0 (0.0)
Type IV	67 (39.9)	66 (38.8)		0 (0.0)	1 (100.0)		67 (40.9)	63 (38.2)		0 (0.0)	2 (50.0)
Type V	23 (13.7)	21 (12.4)		1 (100.0)	0 (0.0)		21 (12.8)	20 (12.1)		1 (33.3)	1 (25.0)
Total	168 (49.7)	170 (50.3)		1 (50.0)	1 (50.0)		164 (49.8)	165 (50.2)		3 (42.9)	4 (57.1)

*D. Vertucci types (N* *=* *338)*												
Type I	161 (95.8)	162 (95.3)	0.857	1 (100.0)	1 (100.0)	NC	157 (95.7)	158 (95.8)	0.881	3 (100.0)	3 (75.0)	1.000
Type II	2 (1.2)	1 (0.6)		—	—		2 (1.2)	1 (0.6)		—	—
Type III	1 (0.6)	2 (1.2)		—	—		1 (0.6)	2 (1.2)		—	—
Type V	4 (2.4)	5 (2.9)		—	—		4 (2.4)	4 (2.4)		0 (0.0)	1 (25.0)
Total	168 (49.7)	170 (50.3)		1 (50.0)	1 (50.0)		164 (49.8)	165 (50.2)		3 (42.9)	4 (57.1)

## Data Availability

The data are available from the corresponding author upon reasonable request.
